# The topology of interpersonal neural network in weak social ties

**DOI:** 10.1038/s41598-024-55495-7

**Published:** 2024-02-29

**Authors:** Yuto Kurihara, Toru Takahashi, Rieko Osu

**Affiliations:** 1 Graduate School of Human Sciences, Waseda University, Tokorozawa, Saitama Japan; 2 Advanced Research Center for Human Sciences, Waseda University, Tokorozawa, Saitama Japan; 3 Faculty of Human Sciences, Waseda University, 2-579-15 Mikajima, Tokorozawa, Saitama Japan

**Keywords:** Cognitive neuroscience, Social neuroscience

## Abstract

The strategies for social interaction between strangers differ from those between acquaintances, whereas the differences in neural basis of social interaction have not been fully elucidated. In this study, we examined the geometrical properties of interpersonal neural networks in pairs of strangers and acquaintances during antiphase joint tapping. Dual electroencephalogram (EEG) of 29 channels per participant was measured from 14 strangers and 13 acquaintance pairs.Intra-brain synchronizations were calculated using the weighted phase lag index (wPLI) for intra-brain electrode combinations, and inter-brain synchronizations were calculated using the phase locking value (PLV) for inter-brain electrode combinations in the theta, alpha, and beta frequency bands. For each participant pair, electrode combinations with larger wPLI/PLV than their surrogates were defined as the edges of the neural networks. We calculated global efficiency, local efficiency, and modularity derived from graph theory for the combined intra- and inter-brain networks of each pair. In the theta band networks, stranger pairs showed larger local efficiency than acquaintance pairs, indicating that the two brains of stranger pairs were more densely connected. Hence, weak social ties require extensive social interactions and result in high efficiency of information transfer between neighbors in neural network.

## Introduction

In everyday life, we experience different levels of intimacy in interpersonal relationships with acquaintances, friends, romantic partners, or strangers. Previous studies have suggested that interacting with people with whom one has stronger social ties, such as romantic partners, family members, and close friends, maybe beneficial^1–4^. People tend to experience less loneliness after intimate interactions^5^. Since the intensity and quality of friendships are positively correlated with life satisfaction^6^, strong social relationships are expected to contribute to well-being. However, in our daily lives, we have many opportunities for social interaction outside our close social groups, such as with strangers or acquaintances with whom we have weak social ties. Interestingly, interacting with people with weak social ties has also been found to contribute to well-being^7–9^. Sandstrom and Dunn (2014)^8^ demonstrated that simply engaging in social interactions with a coffee shop barista (a stranger) can increase people's sense of belonging and well-being.

Although many studies have examined neural responses during intimate interactions, almost no studies have examined interactions in weak social ties. In general, pairs with intimate interpersonal relationships, such as romantic couples and parents, tend to have more synchronized brain activities than pairs with non-special interpersonal relationships. For example, romantic couples (male–female pairs) have been found to have a higher correlation between brain-to-brain electroencephalography (EEG) spectra in gamma band (30–60 Hz) than stranger pairs (male–female pairs) in face-to-face conversations^10^. In the functional near-infrared spectroscopy (fNIRS) studies, romantic partners also had higher inter-brain synchronization than friend pairs during two-handed touching^11^, conversation^12^, and cooperation tasks^13,14^. Parent–child pairs have been found to have higher neural synchrony (fNIRS) than stranger-adult and child pairs during cooperation tasks^15–17^. These studies indicate that intimate pairs exhibit high inter-brain synchronization. In contrast, in the empathy-giving task (sharing their events)^18^, it revealed that weak-tie pairs (strangers and friends) showed higher inter-brain EEG alpha, beta, and gamma band synchronizations with lower behavioral synchronization than romantic couples. However, they showed lower inter-brain EEG synchronization with lower behavioral synchronization than romantic couples in their motor task. Moreover, our previous study showed that weak-tie pairs (including stranger and acquaintance pairs) showed a negative correlation between behavioral synchrony and inter-brain theta EEG synchronization^19^. From these findings, it can be postulated that the relationship between intimacy and inter-brain synchronization is inversely correlated (i.e. complementary) for weak-social tie pairs^18^. Although social interactions between same-gender individuals are experienced on a daily basis, almost no study to date has compared neural synchrony between same-gender strangers and acquaintance pairs. These previous observations of intimate relationships, such as romantic or parent–child pairs, relative to those for weak-tie relationships suggest that neural synchrony is higher in acquaintance pairs than in stranger pairs, owing to the degree of intimacy. However, if intimacy and neural synchrony are complementary, neural synchrony would be higher in stranger pairs than in acquaintance pairs.

Hence, we compared the interpersonal neural networks between stranger and acquaintance pairs during anti-phase joint tapping tasks. Although the task selected is a motor task, it requires the prediction of the movement of the partner. Hence, this task was selected. In addition, compared with in-phase tapping, the anti-phase mode is advantageous in avoiding spurious inter-brain EEG synchronization caused by similarities in movement across individuals. We conducted tests with four tapping conditions: slow (requested inter-tap interval [ITI]:0.5 s), fast (requested ITI:0.25 s), free (preferred ITI), and pseudo (no interaction). To quantify neural synchrony, we performed 29-channel electroencephalography (EEG) and applied a graph-theoretical approach to characterize the topology of the multi-brain network, in addition to comparison using the synchronization indices [Phase Locking Values (PLV) / weighted Phase Lag Index (wPLI )] among EEG channels. For each pair, we created a binary undirected graph consisting of edges (connections between nodes). The topology of the interpersonal neural network can detect the state changes within pairs, such as the demand for musical coordination in guitar duets^20^, social coordination in romantic couples^21^, the degree of flight cooperation in pilot pairs^22^, the difference between joint and solo actions^23^, and emotional communication in parent–child pairs^24^. However, to the best of our knowledge, whether the neural network topology differs when the relations of the pairs differ has not been verified. Thus, we aimed to verify whether neural network topology is related to interpersonal relationships using graph theory.

Fourteen stranger pairs (meeting each other for the first time in an experiment) and 13 acquaintance pairs (one participant brought their partner) participated in the study (data for 19 pairs 〔including stranger and acquaintance pairs〕 were acquired from our previous study data set^19^). To evaluate the synchronization strength, we calculated the wPLI^25^ for intra-brain electrode pairs and PLV^26^ for inter-brain electrode pairs. The reason for using wPLI for intra-brain synchronization was to avoid volume conduction problem^25^. On the other hand, the reason for using PLV for inter-brain synchronization was to use in a lot of studies as a measure to calculate inter-brain EEG synchronization^27,28^. To study the topology of neural network, we compared the graph-theoretical measures including global efficiency, local efficiency, and modularity between stranger/acquaintance groups and tapping conditions. Global efficiency is a measure of the efficiency of information transfer/connectivity across the entire network^29^. Local efficiency is a measure of the average efficiency of information transfer between immediate neighbors of a given network^29^. Both global and local efficiency jointly determine the network’s capability of integrating information effectively^30–32^. Modularity is the degree of independence between two modules^23,33–35^. In this study, we defined two modules as each participant's brain. Thus, high values of modularity characterize situations with independence of connections between two participants' brains. On the other hand, low values of modularity show a high density of connections between them. We focused on theta (4–7 Hz), alpha (8–12 Hz), and beta (13–30 Hz) EEG frequency bands. Several studies have shown that inter-brain EEG theta band synchronization was associated with sustaining cooperative behavior^20,36–40^. Besides, studies examining communication between romantic couples and close friends reported inter-brain beta band synchronization^18,38^. When the cooperative task includes visual attention, inter-brain EEG alpha band synchronization has also been reported^36^. Considering these diverse results, making a clear hypothesis on EEG frequency band is challenging. Therefore, the present study investigated which of these three bands are involved in interpersonal relationship [stranger/acquaintance]. In this study, we did not focus on the gamma band (30 Hz-) since recording gamma oscillations using EEG is fraught with difficulty^39–41^. For example, muscle artifacts may be included in the higher frequency of EEG^42–45^.

## Methods

### Participants

A total of 27 pairs of right-handed participants (13 male pairs, 14 female pairs; mean age = 22.38 years, SD = 2.94 years) were enrolled in the study. Of these 27 pairs, 14 pairs met for the first time in this experiment (defined as stranger pairs), while the remaining 13 already knew each other before participating in the experiment (defined as acquaintance pairs). Two male and four female pairs were excluded from the analysis due to recording artifacts. Thus, 21 pairs were included in the analysis (stranger pairs: 11, acquaintance pairs: 10). The experimental procedures were approved by the Ethical Review Committee of Waseda University and conducted in accordance with the code of ethics of the world medical association (Declaration of Helsinki) for experiments involving humans. All the participants provided written informed consent. Amongst 27 pairs, 19 pairs (strangers: 5 pairs, acquaintances: 8 pairs) were completed from the data samples of our previous study^19^.

### Behavioral task

**Figure 1:**
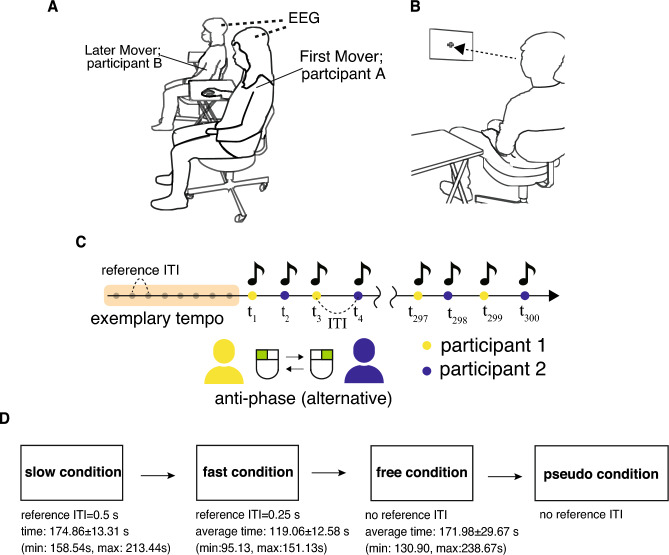
Experimental setups. (**A**) Participants sat side-by-side and wore two wireless electroencephalographs (EEG). (**B**) Each participant gazed at a fixation point in front of him/her during anti-phase tapping. (**C**) The participants performed alternative (anti-phase) tapping. (**D**) We conducted four different tapping conditions: slow, fast, free, and pseudo conditions. These illustrations were created by the first author of the past study^19^ and are used with the permission of all researchers involved in this study^19^, including the first authors.

Participants in each pair were seated side by side (Fig. 1A) and asked to gaze at a fixation point during the tapping task (Fig. 1B). The distance between the participants was approximately 70 cm. Participants were asked to coordinate anti-phase tapping using two computer mice. Each computer mouse was placed on two tables (40 × 50 cm). The participant who tapped first used the mouse’s left click with his/her right index finger. The tapping produced sound feedback (sound frequency of 440 Hz). Each participant listened to the sounds produced by their own taps and their partner’s taps through earphones.

The participants participated in four sessions of anti-phase tapping, with each participant performing 150 taps per session (300 taps per pair) (Fig. 1C). The tapping sessions consisted of four conditions: slow, fast, free, and pseudo conditions (Fig. 1D). In the slow and fast conditions, the participants listened to an exemplary frequency from a metronome, as a reference tempo. After the first eight sounds were transmitted, the metronome was switched off and the pairs started tapping at a frequency as close as possible to the memorized reference Inter-tap interval (ITI) (slow:0.5 s, fast:0.25 s). In the free condition, there was no reference for ITI and the participants tapped at their preferred frequency. In the pseudo condition, after eight sounds of ITI = 0.50 s, the participants continued tapping the metronome (ITI = 0.50 s). We conducted free and pseudo conditions after the slow/fast conditions since for the free condition, no reference was provided and therefore participants would not have understood the task, and for the pseudo condition we aimed to conduct interactive tasks (slow, fast, free conditions) as close as possible to the state of the first meeting in the stranger pairs.

### EEG acquisition

**Figure 2:**
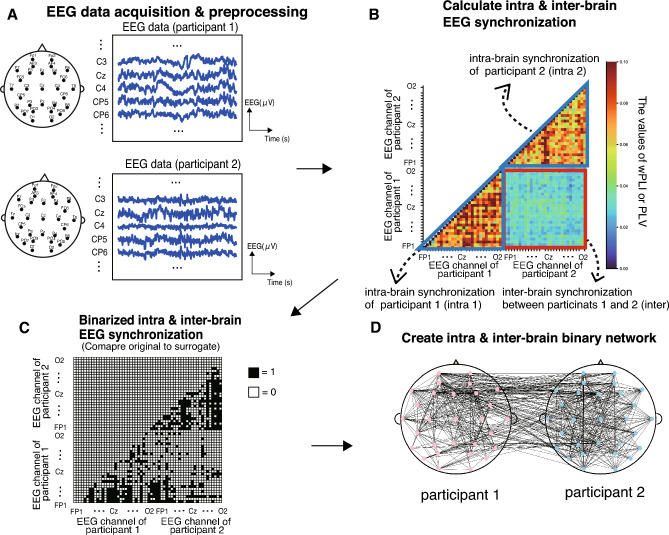
The procedure of intra- and inter-brain electroencephalography (EEG) analysis. (**A**) We collected EEG data from two participants simultaneously. These EEG datasets were filtered into theta, alpha, and beta bands. (**B**) We calculated intra- and inter-brain EEG synchronization. The areas enclosed by the blue triangles show the intra-brain synchronization of participants 1 and 2, respectively. The “intra 1” shows within brain synchronization in participant 1 and the “intra 2” shows within brain synchronization of participant 2. The area enclosed by the red square shows inter-brain synchronization. (**C**) We compared original EEG synchronization to surrogate EEG synchronization and conducted binarization of intra- and inter-brain synchronization (values equal 1 if original synchronization was significantly larger than that of surrogate, else, it equals 0). Note that the upper portion of the matrices were omitted because it is vertically symmetric. (**D**) From the binary matrices of intra- and inter-brain, we created an interpersonal neural network.

We acquired EEG activities from each participant simultaneously. The EEG device had a 29-channel acquisition system (Quick-30; Cognionics, San Diego, CA, USA) in accordance with the international 10/20 system: Fp1/Fp2, AF3/AF4, F3/F4, F7/F8, FC5/FC6, C3/C4, T7/T8, CP5/CP6, P3/P4, P7/P8, PO3/PO4, PO7/PO8, O1/O2, Fz, Cz, and Pz (Fig. 2A). The sampling rate was 500 Hz. A reference electrode was placed on the left earlobe.

### Behavioral analysis

The ITI of anti-phase tapping was calculated by subtracting the tap onset time from next tap onset time. The ITI was defined as follows:

1 $$\begin{array}{c}IT{I}_{m}=t\left(m+1\right)-t\left(m\right) \end{array}$$

2 $$\begin{array}{c}{\text{M}}{\text{e}}{\text{a}}{\text{n}} \, {\text{I}}{\text{T}}{\text{I}}=\frac{1}{M}\sum_{k=1}^{M} IT{I}_{m} \end{array}$$

where $t\left(m\right)$ show the m-th tapping timing including participant 1 and 2, and M is the total number of tapping timings. ${\text{Mean ITI}}$ shows the average of ITI in individual participant pairs.

We calculated the relative phase (RP) of tapping using a circular measure to confirm that the tapping coordination was antiphase (RP = 180°)^19,46,47^. The RP was defined using tapping times:

3 $$\begin{array}{c}R{P}_{n}=\left[\frac{t(n{)}_{p2}-t(n{)}_{p1}}{\left.t(n+1{)}_{p1}-t(n{)}_{p1}\right)}\right]\times 360 \end{array}$$

where $t(n{)}_{p1}$ and $t(n{)}_{p2}$ denote the tapping time (second) of participants A and B, respectively. Here, n denotes the number of tapping for each of participant 1 and 2. The RP ranges from 0°to 360°.

To evaluate the variance from the anti-phase, we calculated the circular standard deviation of RP (SDRP) as follows:

4 $$\begin{array}{c}SDRP=\sqrt{-2{\text{log}}R} \end{array}$$

5 $$\begin{array}{c}R=\left|\frac{1}{N} \sum_{k=1}^{N}{\text{exp}}\left(jR{P}_{k}\right)\right|\end{array}$$

where SDRP represents the instability of anti-phase tapping. The R is the resultant length of the average RP vector. The N is the total number of tapping data points. The j represents the complex number.

### EEG preprocessing

The EEG data were downsampled to 250 Hz and then filtered to eliminate artifacts using a bandpass filter with a range of 1–45 Hz. Visual checks were performed to eliminate problematic EEG channels. The analysis was omitted for bad channels (mean number of bad channels for all participants: 2.42; SD: 1.25). We performed an independent component analysis (ICA) of the EEG to decrease or remove artifacts (electrooculogram, muscular noise, perspiration, and movement). After excluding bad channels and performing ICA, we conducted interpolation using the spline method for bad channels^48^. To extract the theta (4–7 Hz), alpha (8–12 Hz), and beta (13–30 Hz) frequency bands, EEG data were band-pass filtered. EEG preprocessing was conducted using MNE Python (0.20.7).

### Synchrony of each EEG channel pair in the intra- and inter-brain

The wPLI was used to estimate the intra-brain synchrony of each EEG channel pair (Fig. 2B). The wPLI measures the extent to which the difference in the phase angle between two signals is distributed towards the positive or negative parts of the imaginary axis in the complex plane^25,49^. The wPLI enables the estimation of intra-brain synchronization without the deleterious impact of volume conduction and avoids pseudo-synchronization^50,51^. The wPLI ranges from 0 (unsynchronized) to 1 (perfectly synchronized). The wPLI equation is as follows:

6 $$\begin{array}{c}wPLI=\left|\frac{\sum_{t=1}^{T}\left|{\text{Im}}\left({C}_{xy,t}\right)\right|{\text{sgn}}\left({\text{Im}}\left({C}_{xy,t}\right)\right) }{\sum_{t=1}^{T}\left|{\text{Im}}\left({C}_{xy,t}\right)\right| }\right|\end{array}$$

where ${C}_{xy,t}$ is the cross-spectrum density between signals x(t) and y(t) at time t. The T is the total number of sampling points. x(t) and y(t) show the EEG oscillations filtered into the theta, alpha, or beta frequency bands. The Im{・} shows the imaginary operator. Phase angles x(t) and y(t) were determined using the Hilbert Transform. We calculated the intra-brain synchronization in each EEG channel across the other 28 channels (i.e., the total number of channel pairs was ${}_{29}{C}_{2}=406$) for each tapping condition (slow, fast, free, and pseudo-condition) and EEG frequency bands (theta, alpha, and beta bands).

## Synchrony of each EEG channel pair in the inter-brain.

We used PLV to estimate the interbrain synchrony of each EEG channel pair^27,52^ (Fig. 2B). The PLV measures the phase synchrony between the signals. The PLV was used for each pair (i,k) of electrodes between participants A and B. The PLV_i,k_ (synchronization between channel i and k) equation is as follows:

7 $$\begin{array}{c}PLV=\frac{1}{T}\left|\sum_{t=1}^{T}{e}^{j\left({\phi }_{i}(t)-{\varphi }_{k}(t)\right)} \right| \end{array}$$

where ${\phi }_{i}(t)$ and ${\varphi }_{k}(t)$ are the phase angles of the EEG channels of Participants 1 and 2, respectively. T is the total number of EEG sample points. j denotes a complex number. The phase angles were acquired using the Hilbert Transform. The PLV ranged from 0 (unsynchronized) to 1 (perfectly synchronized). We calculated the inter-brain synchronization in each EEG channel of the participants across 29 channels (i.e., the total number of channel pairs was $29\times 29= 841$).

### Calculation the average strength of intra-/inter-brain synchronization.

To evaluate the average strength of intra-brain synchronization, we averaged wPLI of all intra-brain EEG channel pairs (defined as averaged-wPLI). In addition, to evaluate the average strength of inter-brain synchronization, we averaged PLV of all inter-brain EEG channel pairs (defined as averaged-PLV).

### Creation of surrogate datasets and construction of binary undirected graphs from intra- and inter-brain networks

To create binary undirected graphs with channel pairs that have synchrony, the wPLI or PLV of each channel pair was compared with the surrogate and those with values significantly above the chance level were extracted. To generate surrogate data, we applied a Fourier transform to each EEG data for each channel and performed a random permutation of phase values in the frequency domain while maintaining the power of each frequency and applied an inverse Fourier transform. For each individual, EEG channel, and tapping condition, 100 surrogate datasets were created to produce a distribution of the wPLI or PLV values for significance testing. For each channel pair of each participant pair, the wPLI or PLV value obtained from the original data were then compared with the mean of 100 surrogate data using one-sample t-test. All p-values of all EEG channel pairs (1653 pairs, $\alpha =0.05/1653$) were adjusted using the Bonferroni correction for each participant pair. EEG channel pairs with significantly larger wPLI or PLV value than their surrogates were selected as EEG channel pairs for an undirected graph and connected by the edges of value 1 (Fig. 2C). The nodes of the network show EEG channels. For each dyad, experimental condition, and frequency band, we built a binary undirected adjacency matrix as a squared matrix of dimensions (N_ch_p1_ + N_ch_p2_) $\times$ (N_ch_p1_ + N_ch_p2_), which was a single layer 58 × 58 matrix. The N_ch_p1_ represents the number of nodes (EEG channel) of participant 1, and N_ch_p2_ represents the number of nodes of participant 2.

### Graph theory

Binary undirected graphs were created for each participant pair and each tapping condition, utilizing edges (binary connections between nodes) from the comparison between the original and surrogate (Fig. 2D). To synthetically describe the features of functional networks synthetically, we employed multiple measures of functional brain network topology^53^. We calculated graph-theoretical indices for the combined intra- and inter-brain networks. Specifically, we calculated the edge number, global efficiency, local efficiency, and modularity. These graph-theoretical indices were calculated using Network X (version 3.4)^54^.

*Edge numbers* was defined as the number of edges in the intra- and inter-brain graphs. The equation of the Edge numbers $EN$ is as follows:

8 $$\begin{array}{c}EN=\sum_{\begin{array}{c}i,k\in S\\ i\ne k\end{array}}{a}_{ik} \end{array}$$

where ${a}_{ik}$ indicates the connection status between nodes $i$ and $k$: ${a}_{ik}=1$ when nodes $i$ and $k$ are connected and ${a}_{ik}=0$ when they are not connected. S represents the set of all nodes (EEG channels) in the intra- and inter-brain networks.

*Global efficiency* is the average inverse shortest path length between all the pairs of nodes in a network^23,32,33^. The equation for the global efficiency GE is as follows:

9 $$\begin{array}{c}GE=\frac{1}{s\left(s-1\right)}\sum_{\begin{array}{c}i,k\in S\\ i\ne k\end{array}} {a}_{ik}^{-1}\end{array}$$

where ${\text{t}}$ he $s$ is the total number of nodes.

*Local efficiency* represents the efficiency of communication between all the nodes around node $i$ in a network^23,32,33^. The equation of local efficiency $LE$ is as follows:

10 $$\begin{array}{c}LE=\frac{1}{s}\sum_{i\in S}{LE}_{i}=\frac{1}{s}\sum_{i\in S} \frac{\sum_{k,h\in S,k\ne i} {a}_{ik}{a}_{ih}{\left[{PL}_{kh}\right]}^{-1}}{{d}_{i}\left({d}_{i}-1\right)} \end{array}$$

where ${LE}_{i}$ is the local efficiency of node $i$. ${PL}_{kh}$ is the length of the shortest path between nodes $k$ and $h$, which contains only the neighbors of node $i$. ${d}_{i}$ is the number of links connected to a node.

*Modularity i*s the degree to which a network can be separated into a module of nodes with a maximally possible number of within-module links and a minimally possible number of between-module links^33,34,55,56^. We defined each of the two participant networks as the modules. Thus, high values of modularity characterize situations with independence of connections between two participants' brains. On the other hand, low values of modularity show a high density of connections between them. Modularity equation Q is as follows:

11 $$\begin{array}{c}Q=\frac{1}{2EN}\sum_{ik} \left({A}_{ik}-\frac{{d}_{i}{d}_{k}}{2EN}\right)\delta \left({c}_{i},{c}_{k}\right)\end{array}$$

where ${\text{EN}}$ is the number of edges, $A$ is the adjacency matrix of the graph, and ${k}_{i}$ is the degree of node $i$. ${d}_{i}$ shows the number of edges connected to a node $i$. $\delta \left({c}_{i},{c}_{j}\right)$ is 1 if node $i$ and $k$ are in the same module else it is 0.

### Statistical analyses

#### Behavioral analysis

We performed a Welch’s t-test on the mean ITI and SDRP between stranger and acquaintance groups.

#### EEG analysis

Firstly, we calculated a mixed-effect model on the average of intra-brain synchronization (averaged-wPLI) and the average of inter-brain synchronization (averaged-PLV) in the theta, alpha, and beta bands, respectively. The fixed effects were interpersonal relationship (stranger/acquaintance) and tapping conditions (slow/fast/free/pseudo). The random effect in the averaged-wPLI was participant ID. The random effect in the averaged-PLV was pair ID. The mixed-effect models were calculated separately for each EEG band and each brain synchronization strength (intra/inter).

Next, we performed the mixed-effect model on global efficiency, local efficiency, and modularity. The fixed effects were interpersonal relationship (stranger/acquaintance) and tapping conditions (slow/fast/free/pseudo). The mixed-effect models were calculated separately for each EEG frequency band. These random effects were pair ID. These mixed-effect models were calculated using jamovi (2.3.28.0). Statistics of other graph indices (path length and clustered coefficient) are provided in the supplemental information.

## Results

### Behavioral analysis results

We reported the means and standard deviations of the meanITI for each interpersonal relationship and tapping conditions in Table S1. There were no significant differences in meanITI between stranger and acquaintance pairs in slow, fast, or free tapping conditions using Welch’s t-test (slow: *t*_11.69_ = −1.3652, *p*_*adj*_ = 0.594, *d* = −0.618; fast: *t*_13.19_ = 0.470, *p*_*adj*_ = 1.0, *d* = 0.212; free: *t*_18.77_ = −0.248, *p*_*adj*_ = 1.0, *d* = −0.109; all p-values were adjusted using Bonferroni correction).

The means and standard deviations of the tapping variance (SDRP) for each interpersonal relationship and tapping conditions are reported in Table S2. We conducted Welch’s t-test for SDRP between stranger and acquaintance pairs in slow, fast, and free conditions. There were no significant differences in SDRP between stranger and acquaintance pairs in the slow, fast, or free tapping conditions (slow: *t*_17.45_ = −0.084, *p*_*adj*_ = 1.0, *d* = −0.037; fast: *t*_13.91_ = −0.637, *p*_*adj*_ = 1.0, *d* = −0.285; free: *t*_13.91_ = −0.717, *p*_*adj*_ = 1.0, *d* = −0.315; all p-values were adjusted by Bonferroni correction).

### Average intra-brain synchronization strength

First, we calculated the average wPLI (intra-brain synchronization) (defined as averaged-wPLI) for each participant in the theta, alpha, and beta frequency bands. The overall number of objects analyzed was 42 (21 pairs × 2). Average intra-brain synchronization was calculated using the synchronization strengths of all channels. Next, we analyzed the effect of interpersonal relationship (stranger/acquaintance) and tapping conditions (slow/fast/free/pseudo) on averaged-wPLIs in the theta, alpha, and beta bands, respectively. In the theta frequency bands, we found a significant main effect of stranger/acquaintance groups for averaged-wPLI (*F*_1,40_ = 4.926, *p* = 0.032, *Mean*_*stranger*_ = 0.0752, *SD*_*stranger*_ = 0.021, *Mean*_*acquaintance*_ = 0.0657, *SD*_*acquaintance*_ = 0.026). Thus, stranger pairs showed a higher averaged-wPLI than acquaintance pairs in this frequency band. There were no significant main effects of tapping conditions (*F*_3,120_ = 1.409, *p* = 0.244) or interaction effect (*F*_3,120_ = 0.393, *p* = 0.759). In the alpha frequency bands, there were no significant main effects of groups (*F*_1,40_ = 0.514, *p* = 0.477), the main effect of tapping condition (*F*_3,120_ = 0.877, *p* = 0.455 or their interaction effect (*F*_3,120_ = 0.274, *p* = 0.84). In the beta, there were no significant main effects of groups (*F*_1,40_ = 2.751, *p* = 0.105), the main effect of the tapping condition (*F*_3,120_ = 0.785, *p* = 0.505), or their interaction effect (*F*_3,120_ = 0.802, *p* = 0.495). Thus, stranger pairs have greater intra-brain synchronization than acquaintance pairs in the theta bands.

### Average of inter-brain synchronization strength

First, we calculated the average inter-brain synchronization (PLV) for each participant (defined as *averaged-PLV*) in the theta, alpha, and beta frequency bands. A total of 21 participant pairs were analyzed. We also analyzed the effect of interpersonal relationship (stranger/acquaintance) and tapping conditions (slow/fast/free/pseudo) on averaged PLV in the theta, alpha, and beta frequency bands, respectively. In the theta frequency band, we found a significant main effect of tapping conditions (*F*_3,57_ = 7.81, *p* < 0.001). However, there were no main effect of stranger/acquaintance groups (*F*_1,19_ = 3.69, *p* = 0.0700) and no significant interaction effect (*F*_3,57_ = 1.44, *p* = 0.242). Post hoc paired t-tests using Holm correction showed that the average inter-brain synchronization in the fast condition was higher than that in the slow, free, and pseudo conditions (fast-slow: *t*_57_ = 3.281, *p*_*adj*_ = 0.007; fast-free: *t*_57_ = 4.557, *p*_*adj*_ < 0.001; fast-pseudo: *t*_57_ = 3.546, *p*_*adj*_ = 0.004). Post-hoc paired t-tests of other combinations of tapping conditions showed no significant differences (free-slow: *t*_57_ = −1.276, *p*_*adj*_ = 0.621; pseudo-slow: *t*_57_ = −0.265, *p*_*adj*_ = 0.792; free-pseudo: *t*_57_ = −1.011, *p*_*adj*_ = 0.633).

In the alpha frequency band, the factor of tapping conditions showed a significant main effect (*F*_3,57_ = 11.177, *p* < 0.001). The factor of stranger/acquaintance groups showed no main effect (*F*_1,19_ = 0.203, *p* = 0.657). In addition, there was no interaction effect (*F*_3,57_ = 0.317, *p* = 0.813). Post-hoc paired t-tests using Holm correction showed that the averaged-PLV of the fast condition was higher than that in the slow, free, and pseudo conditions (fast-slow: *t*_57_ = 4.925, *p*_*adj*_ < 0.001; fast-free: *t*_57_ = 4.798, *p*_*adj*_ < 0.001; fast-pseudo: *t*_57_ = 4.397, *p*_*adj*_ < 0.001). Post-hoc paired t-tests of other combinations of tapping conditions showed no significant differences (free-slow: *t*_57_ = 0.126, *p*_*adj*_ = 1.00; pseudo-slow: *t*_57_ = 0.528, *p*_*adj*_ = 1.00; free-pseudo: *t*_57_ = −0.402, *p*_*adj*_ = 1.00).

For the beta bands, the tapping condition showed a significant main effect (*F*_3,57_ = 17.25, *p* < 0.001). The factor of stranger/acquaintance groups showed no main effect (*F*_1,19_ = 0.511, *p* = 0.484). In addition, there was no interaction effect (*F*_3,57_ = 1.536, *p* = 0.215). Post hoc paired t-tests using Holm correction showed that the average inter-brain synchronization of the fast condition was higher than that of the slow, free, and pseudo conditions (fast-slow: *t*_57_ = 5.892, *p*_*adj*_ < 0.001; fast-free: *t*_57_ = 6.093, *p*_*adj*_ < 0.001; fast-pseudo: *t*_57_ = 5.594, *p*_*adj*_ < 0.001). Post hoc paired t-tests of other combinations of tapping conditions showed no significant differences (free-slow: *t*_57_ = −0.201, *p*_*adj*_ = 1.00; pseudo-slow: *t*_57_ = 0.299, *p*_*adj*_ = 1.00; free-pseudo: *t*_57_ = −0.499, *p*_*adj*_ = 1.00). Thus, these results suggest that the fast condition of average inter-brain synchronization was the highest among the four tapping conditions in the theta, alpha, and beta frequency bands.

### Geometric features of neural networks

**Figure 3:**
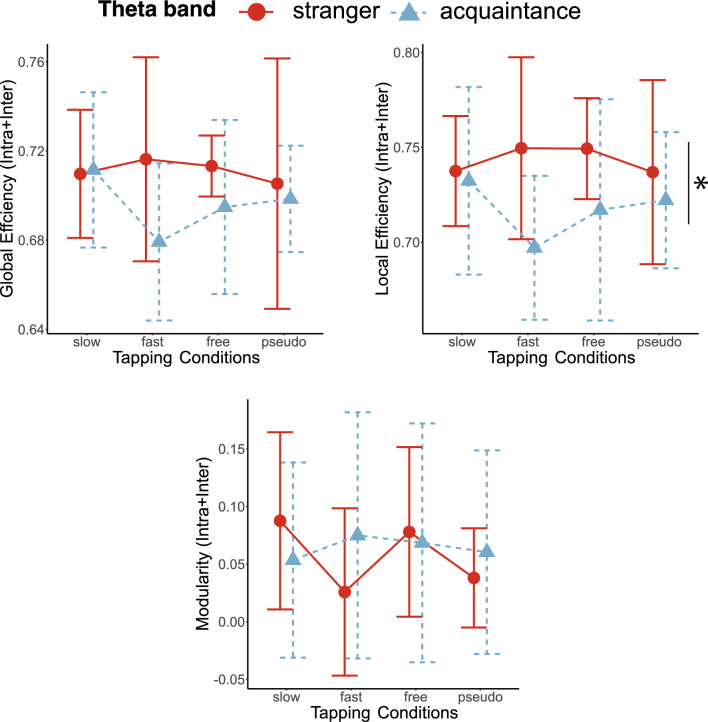
The line plots of graph theory indices of intra- and inter-brain networks in the theta frequency bands in the interpersonal relationship and tapping conditions. There was significant main effect of interpersonal relationship on Local Efficiency in the theta band. The error bars show standard deviations. The asterisk in the figures means the significant differences (p < 0.05).

**Figure 4:**
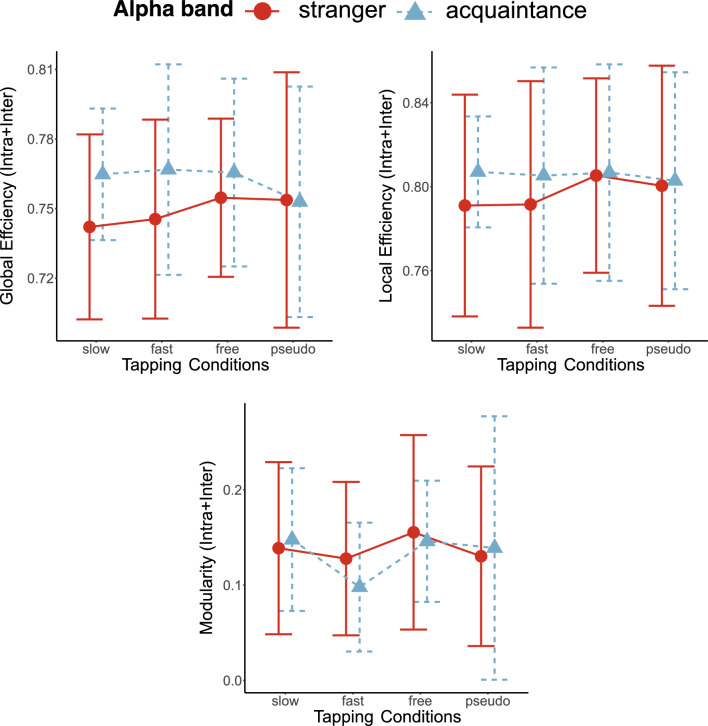
The line plots of graph theory indices of intra- and inter-brain networks in the alpha frequency bands in the partner and tapping conditions. There were no significant main effects of interpersonal relationship and tapping conditions, and interaction. The error bars show standard deviations.

**Figure 5:**
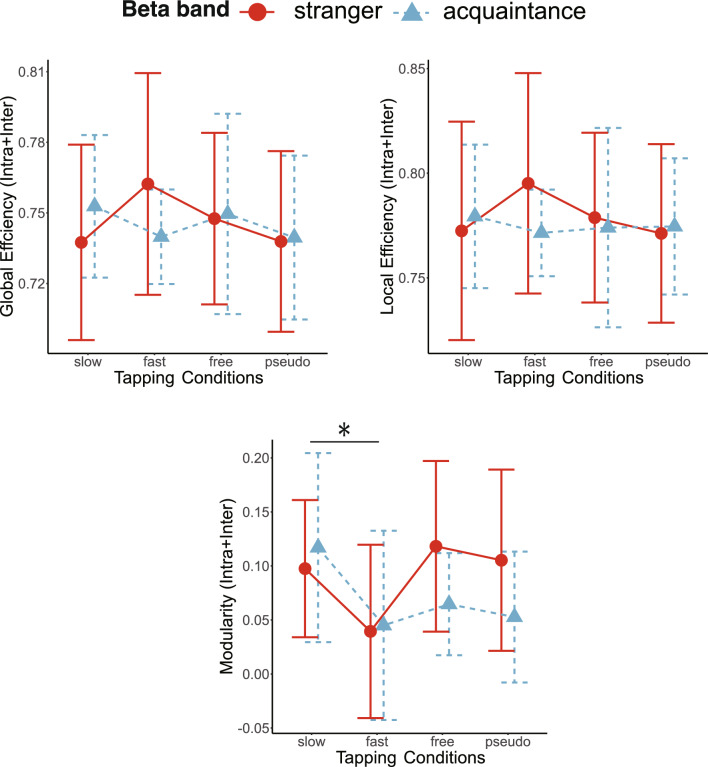
The line plots of graph theory indices of intra- and inter-brain networks in the beta frequency bands in the partner and tapping conditions. There was significant main effect of tapping conditions on modularity in the beta band. The asterisk in the figures means the significant differences (p < 0.05). The error bars show standard deviations.

**Figure 6:**
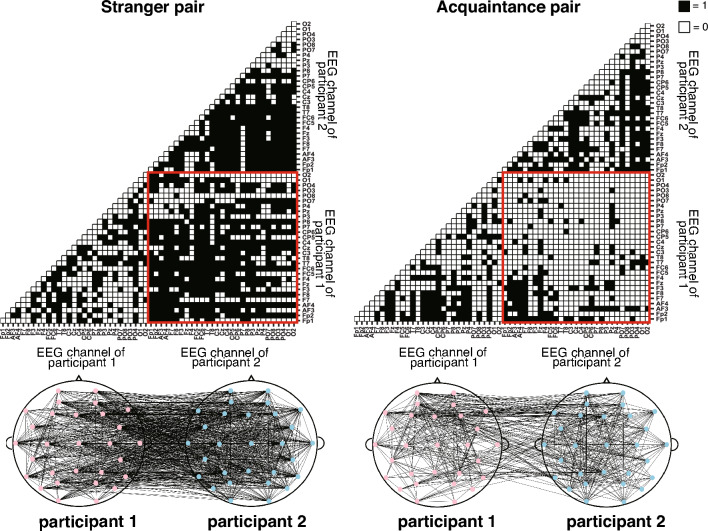
The illustration of matrix and neural network. This is the representative data (fast condition, theta frequency band). The red squares indicate the matrix of inter-brain connectivity. Note that the upper portion of the matrices were omitted because it is vertically symmetric.

The averages of the total number of edges in the combined intra- and inter-brain networks for stranger pairs and acquaintance pairs are reported in Table 1 for each tapping condition and EEG frequency band. We used a mixed-effect model for global efficiency, local efficiency, and modularity of combined intra- and inter-brain networks in the theta, alpha, and beta frequency bands. The results showed a significant main effect of partner for local efficiency in the theta band (Table 2). As shown in Fig. 3, acquaintance pairs showed lower local efficiency than stranger pairs in the theta band. In addition, we observed the significant main effect of tapping conditions for modularity in the beta frequency band (Fig. 5). The post hoc analysis of modularity for the tapping condition showed that the fast condition was significantly higher than the slow and free conditions (fast-slow:* t*_57_ = −3.167, *p*_*adj*_ = 0.015). Post hoc paired t-tests of other combinations of tapping conditions showed no significant differences (free-slow:* t*_57_ = −0.773, *p*_*adj*_ = 0.885; pseudo-slow: *t*_57_ = −1.376, *p*_*adj*_ = 0.522; fast-free: *t*_57_ = −2.394, *p*_*adj*_ = 0.100; fast-pseudo: *t*_57_ = −1.791, *p*_*adj*_ = 0.314; free-pseudo: *t*_57_ = 0.603, *p*_*adj*_ = 0.885; Fig. 4). Others showed no significant differences in the theta, alpha, and beta frequency bands (Figs. 3, 4 and 5, Table 2). Thus, stranger pairs indicated higher local efficiency than acquaintance pairs at theta band. In addition, modularity in the beta band was lower under fast-tapping conditions than other tapping conditions. The examples of stranger/acquaintance neural networks are illustrated in Fig. 6 (the networks of all pairs in the fast condition in the theta band were described in Figs. S1 and S2). In addition, the results of path length and clustering coefficient in the theta, alpha, and beta bands are described in the supplemental information (Table S3).

**Table 1:** The mean and SD of the edge number in the combined intra- and inter-brain network.

	Slow		Fast		Free		Pseudo	
	Mean	SD	Mean	SD	Mean	SD	Mean	SD
Theta								
Stranger	986.09	171.07	1054.45	238.22	999.09	74.99	992.273	248.23
Acquaintance	1011.5	182.83	848.1	198.35	919.5	180.99	942.8	110.39
Alpha								
Stranger	1076.73	159.56	1105.82	185.32	1124.73	131.13	1149.45	249.28
Acquaintance	1181	164.74	1233.9	223.33	1182.1	161.07	1128.9	210.19
Beta								
Stranger	1096.64	202.25	1254.64	225.59	1127.09	173.81	1089.45	176.01
Acquaintance	1154.8	152.94	1147.9	110.11	1178.5	183.49	1136.6	126.15

*SD* standard deviation.

**Table 2:** The results of mixed-effect model for interpersonal relationship and tapping condition at global efficiency (GE), local efficiency (LE), and modularity (Modu), respectively.

	Interpersonal relationship		Tapping conditions		Interaction	
	F (1,19)	p-value	F (3,57)	p-value	F (3,57)	p-value
Theta						
GE	2.715	0.116	0.492	0.689	1.206	0.316
LE	4.630	0.044	0.394	0.758	1.616	0.196
Modu	0.113	0.740	0.564	0.641	1.134	0.343
Alpha						
GE	1.016	0.326	0.179	0.910	0.527	0.665
LE	0.229	0.638	0.197	0.898	0.236	0.871
Modu	0.037	0.851	1.000	0.400	0.317	0.813
Beta						
GE	0.006	0.938	0.575	0.634	1.234	0.306
LE	0.120	0.733	0.356	0.785	0.853	0.471
Modu	0.925	0.348	3.638	0.018	1.737	0.170

## Discussion

 In this study, we examined the differences in interpersonal neural network topology between stranger and acquaintance pairs during a joint-tapping task. There was no difference in the behavioral performance indicated by the variance of the tapping phase between the two groups. We showed significant differences in the average strength of intra-brain synchronization (averaged-wPLI) between the two groups, whereas no significant differences in the average strength of inter-brain synchronization (averaged-PLV) were observed. Besides, there was a significant difference in network topology. Specifically, the neural networks of stranger pairs showed higher efficiency of information transfer between neighbors in the intra-/inter-brain neural network, as indicated by higher local efficiency values. These findings suggest that stranger pairs may engage in a higher level of social interaction than acquaintance pairs during tasks requiring mutual prediction.

We found that the stranger pairs had significantly higher local efficiency in the intra-/inter-brain neural network than acquaintance pairs. In our study, there was a large positive correlation between local and global efficiency in the theta band as well as other bands (Fig. S3). Thus, stranger pairs had more integrated information efficiently in the intra-/inter-brain network than acquaintance pairs. Previous research shows that pairs with strong social ties (romantic partners) display greater EEG gamma bands (30–60 Hz) synchronization when gazing at each other and expressing positive emotion in naturalistic conversation than pairs with weaker social ties (strangers), but not in theta (4-7 Hz), alpha (8-12 Hz), or beta (13-30 Hz) bands^10^. These results suggest the possibility that emotional engagement in pairs with higher levels of intimacy induces greater EEG gamma synchronization. The gamma oscillation is related to social cognition, such as empathy, mentalization, and emotion^57,58^. Therefore, inter-brain gamma EEG synchronization may reflect the social cognition between pairs rather than the behavioral rhythms of joint action. Since we did not analyze the gamma frequency band because of limitations in the measurements, we cannot ascertain whether acquaintance pairs were more synchronized in the gamma band than stranger pairs. However, when participants perform contrived tasks that include joint action, their EEGs tend to converge in a lower frequency band^27,39^. For example, inter-brain EEG synchronization is stronger in the theta and alpha bands during a joint task than when performing the task alone (no joint action)^23^. In addition, theta inter-brain synchronization is related to motor coordination process^57^. Therefore, our results may indicate that brain-to-brain theta synchronization depends on engagement in joint action rather than on social, emotional cognition.

Previous observations may explain why the brains of stranger pairs are more synchronized with each other than those of acquaintance pairs when interaction demand is high could be explained based on previous studies. A dual-fNIRS study has suggested that social interactions with a high mental load induce higher inter-brain synchrony^59^. Furthermore, in socially attentive situations, more mentalization is required, which may induce brain synchronization between each other^60,61^. Cooperation between strangers may produce high mental loading^62,63^. Thus, it is reasonable that stranger pairs were more densely connected to each other in the neural network than acquaintance pairs since stranger pairs were more attentive to the mutual prediction of their behavior than acquaintance pairs. Djalovski et al. showed that in social tasks, inter-brain EEG [alpha (8–12 Hz), beta (13–30 Hz), and gamma (31–48 Hz) bands] synchronization is higher in stranger pairs than in friends or couples^18^. In addition, Kikuchi et al. argued that stranger pairs may show higher inter-brain fNIRS synchronization than acquaintance pairs in an economic exchange task^64^. There may be a nonlinear relationship between the level of intimacy and social engagement, and thus, brain-to-brain synchronization.

We found that interpersonal relationships were associated with lower frequencies (the theta band). It has been shown that the more complex the social interaction, the greater the EEG activity in the theta band^65^. Blume et al. interpreted brain activity in the theta band as reflecting increased demands on the participants' attention and working memory resources when observing complex social interactions. In fact, many studies have shown that theta EEG oscillations play a role in cognitive functions, such as memory encoding and retrieval, working memory retention, novelty detection, and realizing the need for top-down control^66–71^. For stranger pairs, the partner is a more novel social stimulus than for acquaintance pairs. A load of top-down processing and retention of working memory may be higher in the process of tapping when the partner is a stranger than when the partner is an acquaintance^62,63^. Thus, it is reasonable to find an increase in theta-band synchrony related to working memory and top-down processing in stranger pairs.

Our study has three limitations. Firstly, we did not control type 1 error for multiple mixed effect models across the graph theory indices. The present study is exploratory since the functional significance of graph theory indices and EEG frequency bands for brain-brain coupling is still unclear. Based on this result, future studies should be driven by hypothesis and deal with this problem. Secondly, in graph theory analysis, there were only a limited number of methods for selecting important EEG channel pairs. Our selection of significant EEG channel pairs (in comparison with surrogate datasets) to create a neural network for each pair was based on a previous study^24^. There are possible ways of channel selection in group-level analysis, such as selecting EEG channel pairs whose synchronization indices are significantly higher in target tasks (e.g., joint tapping) than in baseline tasks (e.g., independent tapping or resting); however, these do not apply to generating a network for each participant since it requires a statistical test for each channel pair of each participant^19,72^. Thirdly, we did not randomize the different experimental conditions (fixed the order of experimental conditions). Thus, the experimental conditions order and type of condition are confounding factors that cannot be separated.

## Conclusion

To the best of our knowledge, this is the first study comparing the geometric structures of neural networks across interpersonal relationships and coordination. We conducted a two-factor experiment in which the interpersonal levels involved stranger and acquaintance and the levels of interpersonal coordination were slow, fast, free, and pseudo-antiphase joint tapping conditions. We found that, in the EEG theta band, stranger pairs were significant higher local efficiency than acquaintance pairs. This means that the two brains of stranger pairs efficiently integrated information than in acquaintance pairs. In addition, in the beta EEG bands, the modularity was highest in the fast condition. This means that the difficulty of interpersonal coordination was higher than in the other conditions. Our results suggest that weak social ties require extensive social interactions and result in high efficiency of information transfer between neighbors in the neural network.

### Supplementary Information

Supplementary Information.

## Data Availability

The EEG and tapping data generated or analyzed during this study are available at https://osf.io/kgpz2/.
