# Supplementary material for: The topology of interpersonal neural network in weak social ties
Source: Sci Rep. 2024 Feb 29;14:4961. doi: 10.1038/s41598-024-55495-7 (PMC11336176; doi:10.1038/s41598-024-55495-7)
Supplement: Supplementary file 1 — Supplementary Information. [file 41598_2024_55495_MOESM1_ESM.docx]

**The topology of interpersonal neural network in weak social ties**

**Yuto Kurihara^a^, Toru Takahashi^b^, Rieko Osu^c*^**

^a^Graduate of School of Human Science, Waseda University, Saitama, Japan

^b^Advanced Research Center for Human Sciences, Waseda University, Saitama, Japan

^c^Faculty of Human Science, Waseda University, Saitama, Japan

**The additional calculation of graph theoretical measures**

We computed path length and clustering coefficient as well as edge number, global efficiency, local efficiency, and modularity. The path length (PL) is the network's average shortest path length [^1,2^]. The shortest path length between two nodes is the shortest number of edges that must be traversed from one node to the next [^3^]. The equation for the path length PL is as follows:

$$\begin{aligned} \boldsymbol{PL=}\frac{\boldsymbol{1}}{\boldsymbol{s}\left( \boldsymbol{s-1} \right)}\sum_{\boldsymbol{i\neq k}} \boldsymbol{a}_{\boldsymbol{ik}}\boldsymbol{\#}\left( \boldsymbol{S}\boldsymbol{1} \right) \end{aligned}$$

where $a_{ik}$ indicates the connection status between nodes $i$ and $k$: $a_{ik}=1$ when nodes $i$ and $k$ are connected and $a_{ik}=0$ when they are not connected. The $s$ is the total number of nodes.

The clustering coefficient (CC) expresses the degree of connectedness between a node's neighbors. It is defined as the number of triangles that surround a node or the number of neighbors that are neighbors of each other [^1,2^]. The equation for the CC is as follows:

$$\begin{aligned} \boldsymbol{CC=}\frac{\boldsymbol{1}}{\boldsymbol{s}}\sum_{\boldsymbol{i\in S}} \boldsymbol{CC}_{\boldsymbol{i}}\boldsymbol{=}\frac{\boldsymbol{1}}{\boldsymbol{s}}\sum_{\boldsymbol{i\in S}} \frac{\boldsymbol{2}\boldsymbol{T}_{\boldsymbol{i}}}{\boldsymbol{d}_{\boldsymbol{i}}\left( \boldsymbol{d}_{\boldsymbol{i}}\boldsymbol{-1} \right)}\boldsymbol{\#}\left( \boldsymbol{S}\boldsymbol{2} \right) \end{aligned}$$

where ${CC}_{i}$ is the clustering coefficient of node $i$. $T_{i}$ is the number of triangles of the three nodes around node $i$ and $d_{i}$ is the number of edges in node $i$. These results of mixed effect model for interpersonal relationship (stranger/acquaintance) and tapping conditions (slow/fast/free/pseudo) at PL and CC were described in Table S3.

**References**

1. Rubinov, M. & Sporns, O. Complex network measures of brain connectivity: Uses and interpretations. *Neuroimage* **52**, 1059–1069 (2010).

2. Watts, D. J. & Strogatz, S. H. Collective dynamics of ‘small-world’ networks. *Nature* **393**, 440–442 (1998).

3. Sporns, O., Chialvo, D. R., Kaiser, M. & Hilgetag, C. C. Organization, development and function of complex brain networks. *Trends Cogn. Sci.* **8**, 418–425 (2004).

**Table S1: The mean and standard deviation of meanITI (s).**

|  | slow |  | fast |  | free |  |
| --- | --- | --- | --- | --- | --- | --- |
|  | Mean | SD | Mean | SD | Mean | SD |
| stranger | 0.552 | 0.023 | 0.390 | 0.028 | 0.559 | 0.101 |
| acquaintance | 0.579 | 0.057 | 0.382 | 0.055 | 0.570 | 0.102 |

SD, standard deviation.

**Table S2: The mean and standard deviation of SDRP (deg.).**

|  | slow |  | fast |  | free |  |
| --- | --- | --- | --- | --- | --- | --- |
|  | Mean | SD | Mean | SD | Mean | SD |
| stranger | 17.175 | 8.152 | 29.501 | 21.033 | 17.175 | 8.152 |
| acquaintance | 14.459 | 9.121 | 38.028 | 37.315 | 14.459 | 9.121 |

SD, standard deviation.

**Table S3:** The results of the mixed-effect model for clustering coefficient (CC) and path length (PL) of combined intra- and inter-brain matrices in the theta, alpha, and beta frequency bands. These p-values were corrected (adjusted false discovery rate) for four graph theoretical indices.

|  | Interpersonal relationship | | Tapping Conditions | | Interaction | |
| --- | --- | --- | --- | --- | --- | --- |
|  | F (1,19) | p-value | F (3,57) | p-value | F (3,57) | p-value |
| **Theta** |  |  |  |  |  |  |
| CC | 5.091 | 0.036 | 0.099 | 0.960 | 1.043 | 0.381 |
| PL | 2.306 | 0.145 | 0.544 | 0.654 | 1.349 | 0.268 |
| **Alpha** |  |  |  |  |  |  |
| CC | 0.113 | 0.741 | 0.257 | 0.856 | 0.305 | 0.822 |
| PL | 1.059 | 0.316 | 0.203 | 0.894 | 0.486 | 0.693 |
| **Beta** |  |  |  |  |  |  |
| CC | 0.264 | 0.613 | 0.458 | 0.713 | 1.060 | 0.373 |
| PL | 0.004 | 0.950 | 0.578 | 0.632 | 1.206 | 0.316 |


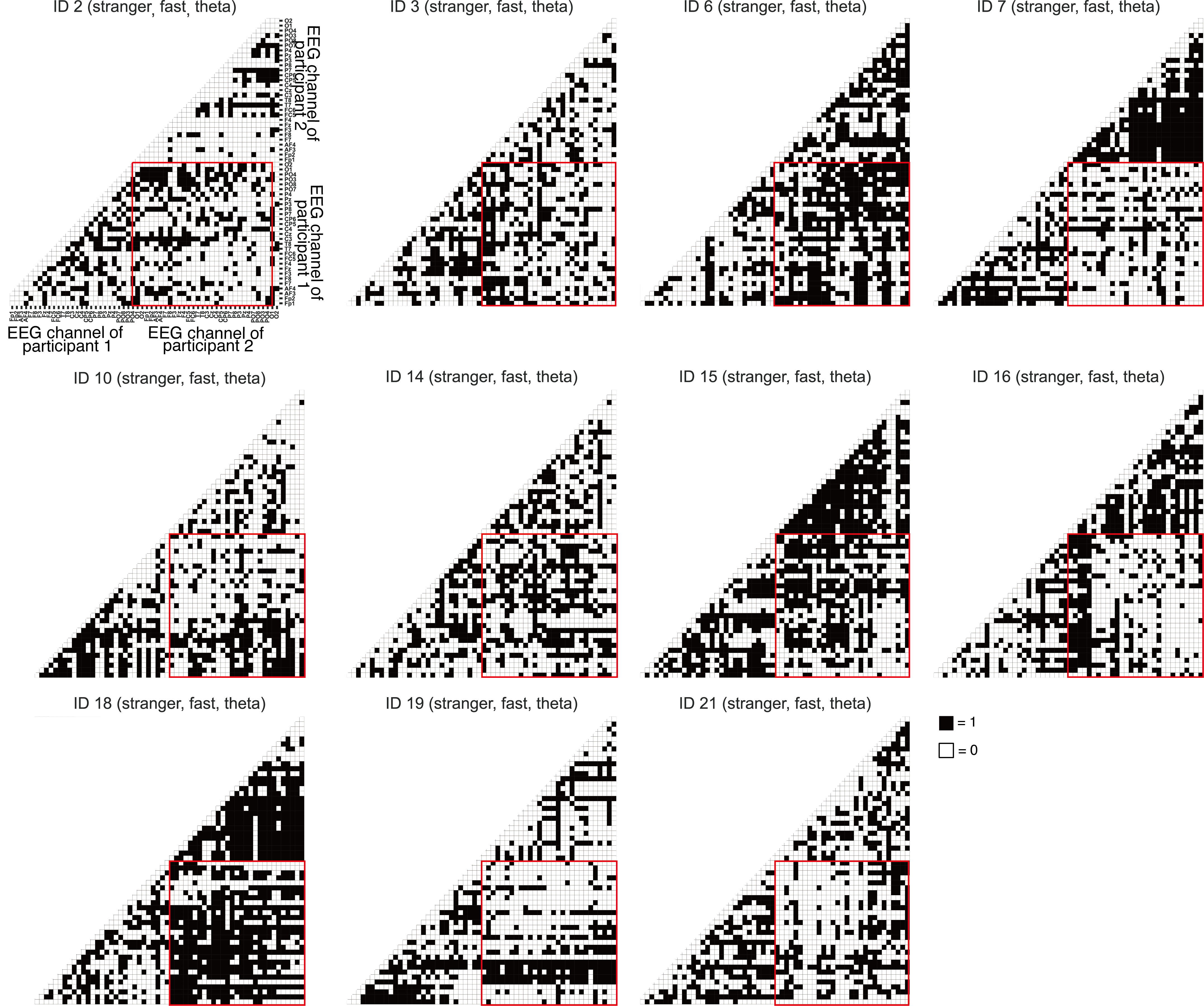


**Fig S1. The illustration of intra- and inter-brain matrices in fast tapping condition in the theta band (stranger pairs).** The red squares indicate the matrix of inter-brain connectivity.


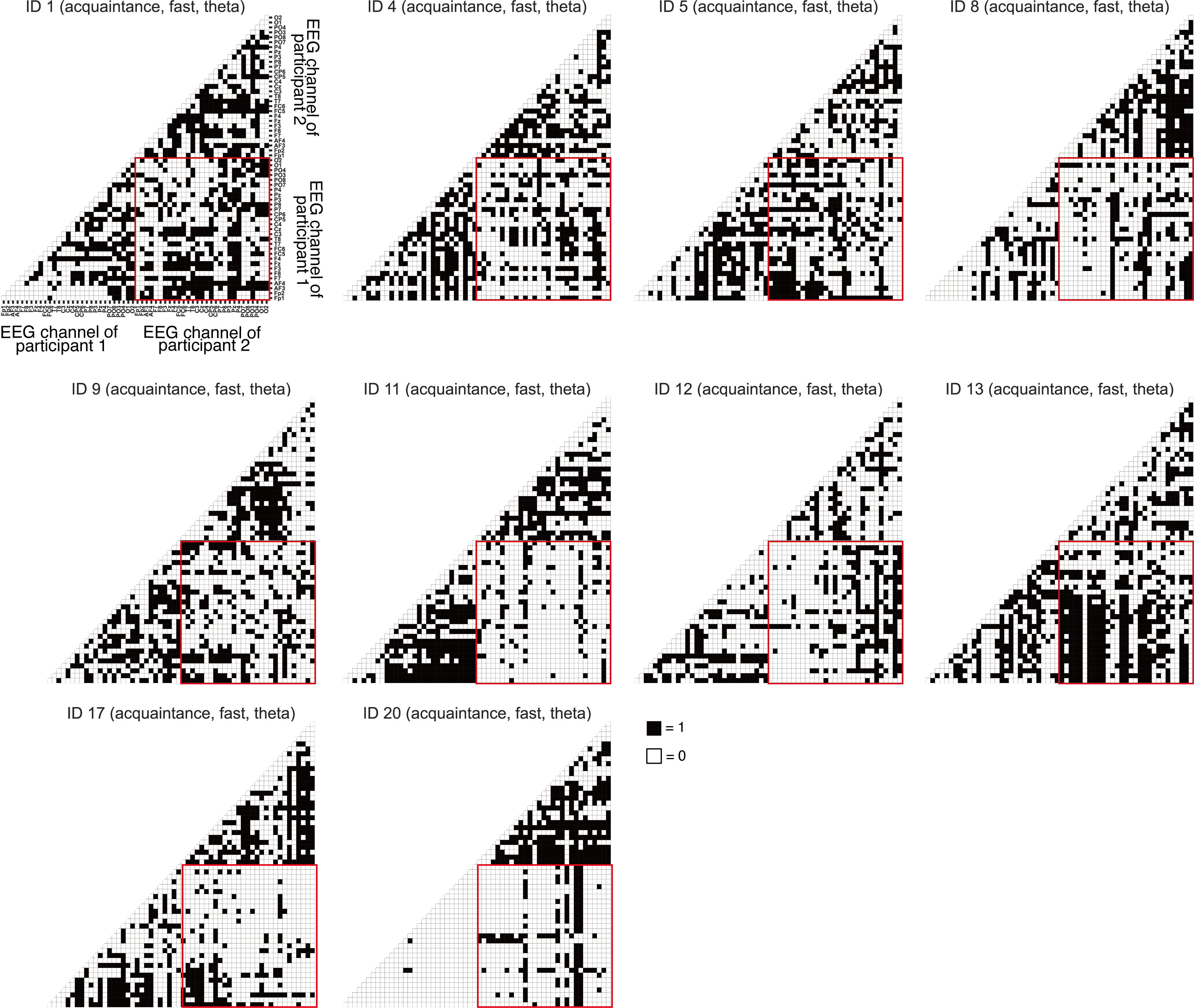


**Fig. S2: The illustration of intra- and inter-brain matrices in fast tapping condition in the theta band (acquaintance pairs).** The red squares indicate the matrix of inter-brain connectivity.


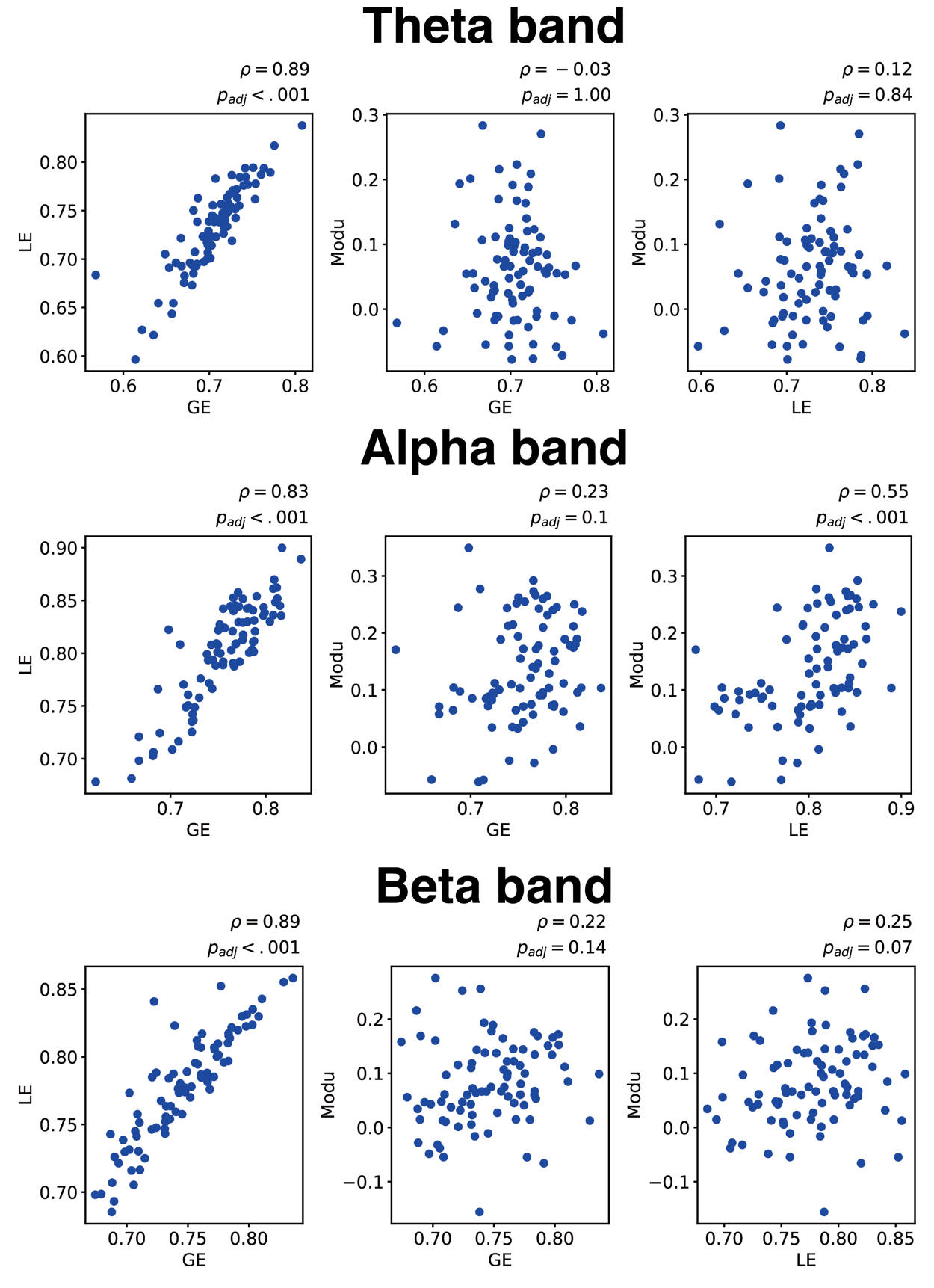


**Fig. S3 The spearman correlations between graph theoretical indices and p-value in the theta, alpha, and beta bands.** The sample data includes slow, fast, free, and pseudo conditions. All p-values were adjusted by Bonferroni correction. GE: Global Efficiency, LE: Local Efficiency, Modu: Modularity
